# ‘Why would they spend all this money and give us these items for free?’: Exploring precarity and power in a cleaner cookstove intervention in rural Malawi

**DOI:** 10.1371/journal.pgph.0001537

**Published:** 2023-02-02

**Authors:** Jane Ardrey, Kate Jehan, Nicola Desmond, Caroline Kumbuyo, Deborah Nyirenda, Stephen B. Gordon, Kevin Mortimer, Rachel Tolhurst

**Affiliations:** 1 Department of International Public Health, Liverpool School of Tropical Medicine, Liverpool, United Kingdom; 2 Department of Public Health, Policy and Systems, Institute of Population Health, University of Liverpool, Liverpool, United Kingdom; 3 Malawi-Liverpool-Wellcome Trust Clinical Research Programme, Blantyre, Malawi; 4 Baylor College of Medicine, Lilongwe, Malawi; 5 Department of Clinical Sciences, Liverpool School of Tropical Medicine, Liverpool, United Kingdom; 6 University of Cambridge, Cambridge, United Kingdom; 7 Liverpool University Hospitals NHS Foundation Trust, Liverpool, United Kingdom; University of Washington Bothell, UNITED STATES

## Abstract

We carried out a qualitative study to gain a deeper understanding of the social context of the Cooking and Pneumonia Study (CAPS) and implications for implementation of clean cooking and similar interventions. Such initiatives are recognised as complex, power-laden processes, which has consequences for outcomes and uptake. However, understanding of how precarious livelihoods and unequal power differentials impact on trials of technology is limited and potentially hampers the achievement of the SDGs including SDG 7, Affordable and Clean Energy. An in-depth exploration of experiences and perceptions of cooking and cookstove use within CAPS was completed using qualitative methods and the participatory methodology Photovoice. Ten CAPS participants from each of five villages participated in Photovoice activities and five village representatives were interviewed. Twelve fieldworkers participated in gender specific focus groups and four were interviewed. A thematic content approach was used for data analysis. The analysis showed that economic and power inequity underpinned the complex social relationships within CAPS impacting on trial participation, perceptions of the cookstoves, and on the potential of the intervention to affect health and other benefits. Power can be understood as relational and productive within the research environment. This is illustrated by an analysis of the role of fieldworkers and community representatives who needed to negotiate resistance to trial compliance decisions, including ‘satanic’ rumours about cookstoves and blood-taking. Transformative approaches that challenge existing power inequities are needed to maximise the success and beneficence of cookstove and other health promoting interventions, and achievement of the SDGs.

## Introduction

Cleaner cooking, which involves reducing air pollution when cooking with biomass fuels, is a key part of the drive to reach Sustainable Development Goal (SDG) 7 and universal access to clean energy by 2030 [1]. Although some progress has been made, for example through solar energy and liquefied petroleum gas (LPG) initiatives, it has been estimated that in 2030 2.3 billion people in Africa and Asia will still be dependent on biomass fuel for cooking [2]. Progress in sub-Saharan Africa (SSA) has been particularly challenging, as population growth between 2000–2018 exceeded growth in the number of people able to access clean cooking [3]. Consequently, it is estimated that 894 million people in SSA, around 85 per cent of the population are exposed to cooking related air pollution and concerted action is required to tackle this ‘”access deficit”‘ [3].

Enabling universal access to clean energy has been linked by the United Nations with a range of other SDGs and goals such as, ‘poverty eradication; food security; clean water and sanitation; health; education; prosperity; job creation; and the empowerment of youth and women’ [4]. The inefficient use of biomass fuels for cooking, on open fires or inefficient cookstoves, also contributes to greenhouse gas emissions and therefore the climate emergency [5].

Cleaner burning cookstoves have been promoted and distributed as part of clean energy initiatives but despite many potential benefits achieving sustained uptake is complex and challenging [6]. The concept of the ‘energy ladder’ suggests a linear pathway from biomass cooking using open fires, through to clean (at the point of use) electric cooking. However, in practice cooks often use fuels and cooking methods interchangeably and may also abandon new technology over time [7]. Despite these issues, in the absence of cleaner cooking solutions such as electricity in many contexts, due to economic constraints, conflict and fragility [8], it seems likely that cookstoves will remain part of clean air strategies utilised in efforts towards the achievement of the SDGs, especially SDG7 [3, 6].

The Cooking and Pneumonia Study (CAPS) was a cluster randomised controlled trial initiated to compare the effect of the use of a cleaner burning cookstove on incidence of pneumonia in under-fives in two rural Malawian communities, Chikwawa in the south and Karonga in the north [9]. The health risks of air pollution have been widely reported and it is estimated 3.8 million premature deaths per annum result from the indoor burning of biomass fuels for cooking and heating [5].

In the CAPS intervention villages, participating households were provided with two forced draft biomass cookstoves with an integral fan powered by a solar panel charged battery, and control village households continued with open-fire cooking. In both arms, data were collected over a two-year period [9]. Further details of the intervention cookstove can be found in the Clean Cooking Alliance Catalog [10]. The trial found that there was no reduction in the incidence of pneumonia and the authors suggest that there were potentially multi-factorial reasons for these results including exposure to ambient air pollution, and limited efficacy and inconsistent use of cookstoves in a real-world setting [9]. Gaining a deeper understanding of how cookstove and similar interventions impact on and are shaped by contextual power relations can help interpret the results of such initiatives. Importantly, they can also contribute to ethical decision making about their suitability and beneficence.

There is a potential window of opportunity, a ‘critical juncture’ [11], provided by the realisation that challenging the power asymmetries in global health, is not only fairer but may be more effective [12]. The United Nations have called for a decade of action in the run up to 2030 as progress towards meeting all SDGs is advancing too slowly to achieve their aims [13], and in a review of SDG7 Affordable and Clean Energy it was reported that the target will be unachievable without a change in strategy [3]. The Global Health 50/50 collective have suggested that the ‘skewed distribution of power, privilege and priorities’ is stymieing achievement of the SDGs, concluding that extending representation and diversity in global health is needed for equitable and healthy societies [12].

Some studies have explored issues of power within similar contexts, that is, clinical trials in highly researched environments [14], often with an emphasis on the role of intermediaries such as fieldworkers [15] and community liaison volunteers [16, 17]. However, the role of power relations in cookstove interventions remains unexplored. Similarly, while it has been suggested that rumours of satanic intent and blood taking by researchers are forms of resistance that are linked with unequal power dynamics in research initiatives in sub-Saharan Africa [18–22], the role of such rumours in cookstove interventions has not previously been examined.

This manuscript is based on the PhD research of the first author (JA) who aimed to gain a deep understanding of the social context of the Cooking and Pneumonia Study. The supposition that underpins this exploration is that ‘techniques of power’ [23] are integral to many health based interventions, particularly in contexts of livelihood insecurity. The approach to power taken in this manuscript is Foucauldian, that is, power is seen as ubiquitous and found in all social relations where it is not only repressive but can be positive and productive [24]. The productive nature of power relates to how it has the potential to create and change, social relations and practices [25]. Therefore, exploring and reflecting on how power was enacted and resisted within CAPS can contribute to ethical decision making about whether and how similar interventions should proceed. Further findings related to understandings of health and gender within CAPS are reported elsewhere [26, 27].

## Study setting and methods

### Study setting

CAPS was carried out in Malawi where in 2018 it was estimated that more than 50% of the population were living in poverty and over 20% in extreme poverty [28]. The majority of the population are employed in an insecure and low-productivity agricultural industry with limited options for other paid work [28]. According to the Malawi 2015–6 Demographic and Health Survey only 4% of rural households had access to electricity and 98% of Malawian household relied on biomass fuel for cooking [29].

The base for CAPS was the Malawi-Liverpool-Wellcome Trust Clinical Research centre (MLW) field site in Chikwawa. MLW was founded in 1995 with an initial focus on malaria and in the next 20 years research activities expanded to include a wide range of topics including lung health [30]. The study was conducted within a community previously exposed to other health interventions. Immediately prior to CAPS the MLW ACTia clinical trial was conducted in the Chikwawa district to explore the safety of repeated malaria treatment on young children [31]. The Chikwawa district is rural, low-lying in the Shire Valley, and prone to seasonal flooding and food shortages [32].

This qualitative study was nested within CAPS in the Chikwawa district and carried out between April and November 2016 towards the end of the clinical trial. Although RCTs are commonly considered to be the ‘”gold-standard”‘ way to evaluate interventions [33], this approach has been criticised as neglecting the “messiness” of the world outside the laboratory [34]. Qualitative research can complement and help elucidate the findings of RCTs by facilitating a deeper understanding of these processes [33].

The participatory methodology Photovoice was included in the study design to explore the in-depth perceptions of CAPS participants and because it explicitly aligns to principles of inclusivity and equity. Photovoice was developed in the 1990s by researchers Wang and Burris to explore the ‘everyday health and work realities’ of Chinese rural women [35]. The roots of the methodology are in the work of Freire and his use of the visual image as a focus for discussion and critical thinking [36], ‘feminist theory and practice’, and documentary photography [37]. By giving people cameras to record their own strengths and concerns, Photovoice recognises that everyone is an expert on their own situation and can provide valuable insight that outsider researchers lack [37].

A pilot Photovoice study was conducted which accentuated the ability and willingness of CAPS participants to share the strengths and challenges of their lives [38]. In this study, Photovoice methodology was used in combination with focus groups, interviews and observation qualitative methods as shown in Table 1.

**Table 1:** Overview of research study.

Date	Research activities
**April 2016**	Observation of cooking activities in Villages 1 and 5
**July 2016**	Observation of cooking activities in Villages 2, 3 and 4
	Photovoice training and image collection in Villages 1–5
	Photovoice participant focus groups in Villages 1–5
	Female and male fieldworker focus groups
**November 2016**	Semi-structured interviews with 15 CAPS participants
	Semi-structured interviews with 4 fieldworkers
	Semi-structured interviews with 5 CoLT members

For the observation and Photovoice elements of the study and in close collaboration with the CAPS field team, five CAPS villages were purposively selected using criteria such as access to trading centres and facilities, size and geographical location to create a maximum variation sample. Within each village, observation sessions were conducted with female cooks purposively selected so that the group included female heads of households and households of varied economic status. In recognition of the primary role of women as household cooks in this context, gender was the primary selection criterion for the Photovoice sessions. Other selection criteria included age, marital status and occupation. See Table 2 for details of villages and Photovoice participants.

**Table 2:** Village characteristics and details of Photovoice participants.

Village	Characteristics	Participants
**1**	• 137 households	8 female, age range 24–32
	• Reasonable access via side road close to main trading centre and M1 road	2 male, age range 28–50
	• School and retail shops	List of stated occupations:
	• Many people employed as sugar cane co-operative shareholders (Kasinthula), growing sugarcane on behalf of the Illovo Company	• Shareholder at Kasinthula co-operative (2 female and 1 male)
		• Mini-bus conductor
		• Sells rice
	• Through Fairtrade (sugar) some homes and the school have electricity	• Subsistence farmer (3 as primary occupation and 1 as secondary)
**2**	• 105 households	8 female, age range 20–65
	• Distant from main trading centre (approximately 1 hour by bicycle taxi)	2 male, age range 23–32
	• No school, 4 small churches	List of stated occupations:
	• Most employed as farmers, some charcoal sellers	• Runs bicycle hire business
		• Casual labourer
		• Subsistence farmer (7 as primary occupation and 3 as secondary)
**3**	• 402 households	8 female, age range 23–38
	• Located just off M1 near trading centre and on the banks of the Shire River	2 male, age range 46–51
	• Secondary school, 3 retail shops, 3 churches, 1 health post (charitable)	List of stated occupations:
		• Sells Doughnuts
	• Most people farm including commercially utilising riverbank location, some fishing	• Sells maize
		• Builder
		• Guard/Pastor
		• Subsistence farmer (6 as primary occupation and 2 as secondary)
**4**	• 90 households	8 female, age range 18–35
	• Close to village 2 –distant from trading centre	2 male, age range 30–36
	• Primary school, maize mill (diesel), 2 churches	List of stated occupations:
	• Most residents are famers	• All–primary occupation of subsistence farmer
		• Shop owner
		• Charcoal burner
**5**	• 180 households	8 female, age range 20–49
	• 1 church and 2 shops	2 male, age range 23–53
	• Alongside M1 but further from trading centre than Village 3, situated on bank of Shire River	List of stated occupations:
		• Sells doughnuts
	• Most people farm including commercially utilising riverbank location, some fishing	• Shop owner
		• Pump attendant (Kasinthula)
		• Sells phones
		• Commercial farmer
		• Subsistence farmer (6 as primary occupation and 3 as secondary)

Throughout MLW research activities, the role of village CoLTs is key, and in each of the five selected villages they played an active role in liaising with CAPS fieldworkers to set up Photovoice sessions and other research activities for this study. In recognition of their important role as intermediaries between research and community, these village CoLTs were invited to participate in semi-structured interviews. CAPS fieldworkers were also invited to take part in gender specific focus groups and follow up interviews.

### Methods

In overview, in April 2016 two observation sessions were carried out in villages 1 and 5. In July 2016 the following activities took place: Photovoice training and focus group sessions in each of the five villages; two gender specific fieldworker focus groups; observation sessions in villages 2, 3 and 4. In November 2016, four fieldworkers, five CoLT members, 3 observation participants and 12 Photovoice participants were interviewed.

Observation of the cooking of lunch by a female CAPS participant took place in each of the five villages, with JA and CK in attendance. For Photovoice, in each village, sessions were held with the 10 recruited Photovoice participants, eight women and two men, to train them in camera use and image collection. The camera was a basic digital camera with a viewfinder and disposable batteries, and the 50 participants had five days to collect 50 images about all aspects of cooking. They also took 20 images for themselves. Photovoice activities were informed by a previous pilot study [38] and underpinned by key features of the methodology. This included the inclusion of Photovoice participants in foundational analysis though a process of selecting images to be discussed, contextualising them through telling stories about their meaning, and contribution to the codification of emerging issues and themes [37]. An adapted version of the SHOWeD acronym developed by Wang and Burris was used to deepen discussion at the contextualising stage, that is: ‘What do you **S**ee here?; ‘What is really **H**appening here?’; ‘How does this relate to **O**ur lives?’; **W**hy does this situation, concern or strength exist?’; What can we **D**o about it? [35].

In practice, village level focus groups were carried out with each Photovoice participant selecting from their own images (hard copies were provided), those that they wanted to discuss. These were then spread out on large sheets of paper on the floor and grouped together into subject areas, for example, sharing a meal with family members. While the photographer often took the lead by referring to a particular image, discussion usually developed to include other participants and covered a wide range of cooking related issues such as food insecurity and household roles. Follow-up interviews explored any health impacts of the intervention cookstoves and the uses of time saved from faster cooking.

Semi-structured interviews with each of the village CoLT members, two women and three men were also carried out. These were all experienced in their role and village residents and interview topics covered the impact of CAPS on trial participants and local definitions of good health.

A group of fieldworkers volunteered to participate in gender specific FGDs. Although they were not purposively selected, individuals were of various ages and seniority and with eight males and four female fieldworkers, the groups were reflective of the gender balance of the CAPS fieldworker team. In the FGDs fieldworkers discussed: any benefits of CAPS for participants; how CAPS differed from other research they had been involved in; barriers and motivators of adoption of the intervention cookstoves. From the initial group of 12, four fieldworkers, two women and two men, all aged 30–40 volunteered to be interviewed to: explore in more detail potential benefits of cookstoves, cookstove use and gendered household roles.

### Data collection and analysis

The first author (JA) co-ordinated all field research activities, was present throughout data collection except for two fieldworker interviews and analysed the data. The study Research Assistant (CK) led on the selection of participants, carried out two fieldworker interviews and provided translation throughout data collection and analysis.

All FGDs and interviews were recorded; translation and transcription were completed by CK with the support of expert staff from MLW. Thematic framework analysis was used as follows: JA gained familiarity with each transcript, carried out coding to develop a framework, summarised data and collating codes to develop categories and finally mapped and interpretated the data [39]. The emphasis throughout the process, in line with the constructivist approach, was to pay attention to the lived experiences and perceptions of participants, therefore coding was data driven [40] with more conceptual analysis occurring at the interpretation stage. In line with the emphasis within Photovoice on images not standing alone but only being analysed and disseminated in conjunction with participant text [37], images did not form part of the data set. The contribution of Photovoice to the richness of data was encapsulated in FGD and interview discussions as summarised by Wang in an early Photovoice publication:

*‘*People merely creating images is not the key to photovoice, however. The process also requires that people define these images. Photovoice entails people’s discussing their images that they have produced, and by doing so, they give meaning to, or interpret, their images’ [35].

An example of the importance of paying attention to the interpretation and intended meaning of the Photovoice participant is illustrated by the discussion of an image which on first viewing appeared to show a group of children playing. However, the photographer explained that the children were breaking up small fruits because they were hungry (see full quote in the Results).

In summary, images were not analysed or coded but the discussions of images in FGDs and interviews were key to data collection and analysis. A sub-set of Photovoice images were selected by Photovoice participants for display and discussion in and around participating CAPS villages, and images from this sub-set were also displayed in the UK.

### Ethics and consent

Ethical approval was obtained from The Malawi College of Medicine Research Ethics Committee (reference number P.11/12/1308) and the Liverpool School of Tropical Medicine Research Ethics Committee (reference number 12.40). These permissions include the use of images.

## Results

We found that decisions about whether to participate in CAPS, perceptions of cookstoves and the wider trial, and possibilities that trial involvement would improve participants lives, were dependent on complex social relationships underpinned by power and socio-economic inequities. CAPS participants clearly expressed their concerns about equitable access to food through Photovoice activities, highlighting their precarity and their reliance on MLW as a provider of health and other benefits. Uncertainties about the motivations of researchers and the research ecosystem in general were articulated through rumours linking cookstoves and blood-taking. Fieldworkers and CoLT members provided an interface between MLW and CAPS participants, which involved negotiating and moderating these, and other challenges to trial “compliance”.

These findings will be presented through three interlinked themes: Livelihood precarity amplifies power/knowledge inequity; Power relations between CAPS trial actors; Power and resistance expressed through rumours of blood-taking.

### Livelihood precarity amplifies power/knowledge inequity

This analysis demonstrated how the precarity of CAPS trial participants, their day-to-day struggle for individual and household survival in a context of insecure employment and livelihood opportunities, amplified the influential inequitable power/knowledge relationship between MLW as a research institution and its intended research subjects. Swidler and Watkins (2009) used survey, interview, and ethnographic data to examine how initiatives to empower rural Malawian communities impacted on three groups: participating villagers, ‘insecure local elites’ (analogous to fieldworkers), and ‘national elites’ who implemented programmes [41]. Although the authors explored AIDS policy and programmes, their analysis has a wider reach in that they suggest that where marginal agriculture is combined with insecure employment, development initiatives engender a type of ‘hunting and gathering’ where benefits are sought and can ensue, from involvement in such activities [41]. In our research context, livelihood precarity is also reflected in concerns about access to sufficient nutritious food to allow children to thrive, and for adults to participate in demanding physical labour.

In common with Jerneck and Olsson’s (2014) finding that there is a ‘”food imperative”‘ that hampers new agroforestry practices, CAPS participants’ narratives demonstrated how ‘the over-riding task of putting food (and water) on the table’ [42], dominated the cooking context. In all village Photovoice FGDs, when sorting and discussing images about cooking, participants made clear the importance of growing food and particularly maize; the staple food in Malawi is *nsima* made from maize meal. A female FGD participant explained that:

‘[Here] we are also able to see *nsima*. We depend on *nsima* so much here, here in Malawi because it gives us energy [that helps] with the field work that we do, so, we wanted to explain a lot about *nsima*.’ (Female Photovoice participant Village 1 FGD)

The cultivation of maize, and other farming activities entails considerable effort and, in this context, *nsima* is both an end product and a source of energy for arduous manual labour. Dietary protein when available consists mainly of beans or locally caught field mice, birds or fish, with limited consumption of meat.

Many Chikwawa residents grow their own food either on land they rent or own, or in gardens close to their homes. Renting land may not always be economically viable as described by a female Photovoice participant; annual rental was $28 but only three bags of maize were harvested, enough for 3 months [43]. Agriculture is very important to Chikwawa households but is seen as potentially unreliable due to the impact of unpredictable weather on harvests. Many lack the capital, in the form of land, equipment or funds to make farming worthwhile and other livelihood options are limited, adding to uncertainty and a cycle of day-to day survival. In Chikwawa, skilled and dependable income-generating opportunities are largely inaccessible, a type of ad-hoc casual labour *ganyu* predominates [44, 45], and people depend on mixed livelihoods at the margins of survival.

Female photovoice participants reported that they often stayed close to home to make “money for salt”, (meaning to earn money for everyday expenses), through selling doughnuts or dried fish while their husbands seek casual work away from home. However, in lower-income households, both men and women are employed in *ganyu* work, as described by this interviewee:

‘[My husband] does piecework because I shouldn’t lie that he has anything, because we don’t even own goats. We don’t even have cows. We don’t have anything. Yes, both of us do business, someone goes somewhere, the other one goes to a different place, we then meet in the evening.’ (Female Photovoice and Observation Village 2)

Concern about equitable access to food, was also a subject of discussion in Photovoice activities. Participants emphasised making sure that children receive adequate nutrition to allow them to thrive. For example, separate plates, if affordable, were described as a solution for managing the behaviour of younger children whose hunger drives them to take more than their share from communal serving dishes. (Female Observation IDI).

The dominance of concern about childhood hunger is shown in the discussion of an image of a group of children selected by a Photovoice participant as one he particularly wanted to talk about. He said:

‘I can say that all of these pictures interest me because I was taking them with a reason… because if you heard clearly, I said that a lot of people only had vegetable relish [stew eaten with maize meal]. It is the issue of poverty that is making us not to be changing… but there is a picture right here. I took that picture, if you can see clearly the children were hungry that time. So, if you can see the children clearly, there is mfula, do you know mfula? Yes, certain fruits, they break and eat. Yes, so this picture interested me because this was happening because of poverty just because of the season… when I came close to the children and saw what they were doing that time, aaa I felt sympathy.’ (Male Photovoice Village 1).

Within this context of scarcity CoLT members clearly articulated their role as a job, as a possible solution to their financial security. A male CoLT member explained that being a volunteer for MLW is one of the different jobs he does which includes growing food and made a plea for further research projects to come to his village. Similarly, a female CoLT member said that she earns money through her CoLT role and described how her tasks include managing dissatisfaction with differing recompense schemes used in research studies. These findings are concurrent with Geissler’s (2013) supposition that while the poverty and vulnerability of research participants and facilitating staff are well recognised in global health, an emphasis on autonomy as foundational to ethical research obscures the existence and impact of material differences, on the research process [46]. This ‘unknowing’, that is, the lack of acknowledgment of these differences in research texts and public speech, not only allows research to function but shapes how it is conducted and contributes to the maintenance of material inequality [46]. This is illustrated by the dependence on MLW which extends beyond the household to the community, as demonstrated in a description of Chiefs giving village residents ‘pressure’ to participate in research studies and how they ‘mobilize’ residents:

So, some of the Chiefs, it’s like their culture they say, “OK, if you are not participating in this study then if another one comes then you will not be able to participate as you refused this one”. So, it’s their tradition, it’s there, I think nobody can stop that, it will still be there. (Female fieldworker)

In their study of community engagement within a malaria vaccine trial in Kilifi, Kenya, Angwenyi et al. (2014) also found incidences of chiefs and elders using their authoritative power to persuade community members to participate, despite trial voluntary consent processes [47] suggesting that, as with CAPS, such practices are found in contexts of scarcity.

In summary, the ubiquity of livelihood precarity within CAPS villages appears to have encouraged a reliance on MLW, both by individuals and the community collectively, as a source of material and other benefits. In addition, there are indications that the social relations within research infrastructure and process has unintended effects due to ‘the gaps between expectations and understandings of different participants’ [48]. These results also demonstrate the clear power inequity between the research participants and representatives of MLW. In the next section, these power relations will be explored with reference to the challenges encountered by CAPS fieldworkers and CoLTs.

### Power relations between CAPS trial actors

These results suggest that since the possibility of a positive trial outcome depended on specific daily behaviours by trial participants and that field workers and CoLTs were either explicitly or implicitly expected to facilitate such behaviour change. This was constructed within the trial as “correct” use of the cookstoves that impacted on data quality required by researchers. The main explicit way that this was supposed to be done was “sensitization”, that is giving information about the benefits of the stoves, as well as visiting every 3 months. However, for multiple reasons, for example the need to chop fuelwood into small pieces to fit the intervention cookstove combustion chamber, participants did not necessarily use the cookstoves consistently, so it implicitly became the responsibility of fieldworkers and CoLTs to try to change this situation. They describe doing so through multiple tactics, leveraging authority through their formal roles in the network of power created by the material and social relations of the trial and wider MLW research infrastructure.

Fieldworkers suggested that CAPS participants did not always use the cookstoves ‘according to the protocol’ (Female fieldworker). The challenge of CAPS, according to fieldworkers was that it involved participants carrying out specific behaviour several times a day, as opposed to a more conventional clinical trial where trial procedures are intermittent and largely based in clinical settings. Although fieldworkers recognised the difficulty of changing long-standing cooking behaviour, this was seen as necessary ‘to improve the quality of the data’. (Female fieldworker) That is, CAPS as a randomised controlled trial depended on the consistent use of cookstoves in the intervention households to allow a comparison with the status quo.

These difficulties were managed through additional visits and communication, to explain why the cookstoves had been provided and the responsibility of participants regarding stove use; this “sensitisation” needed to be done ‘time and time again so it was tough going’ (Male fieldworker). A specific example related to the 3-month household visits to check that cookstoves were being used as set out in the protocol. The fieldworkers discovered that in anticipation of these, some participants would get out their cookstoves and ‘start polishing them up’ (Female fieldworker), that is, they brought them out of storage and began to use them. However, fieldworkers were able to take advantage of a misunderstanding about stove use monitors (SUMS), a device added to a sub-set of the cookstoves to record periods of use. Some cookstove users assumed that the SUMS reported use in real time, ‘there was a rumour that it speaks’ whereas in actuality data was downloaded when the SUMS was collected (Female fieldworker). That is, they were able to utilise information asymmetries to strengthen the disciplinary regime that was created by educational inequities. Other strategies were also employed such as suggesting that visits would be made at random times and asking CoLT members to check on use.

This CoLT supervisory role is clearly described below:

The study went very well because I was able to monitor the participants. Yes, I played a big role…because I was able to do what the bosses at office would want me to do. At first people were not eager to use the cook stoves [but] then I started monitoring them and encouraging them…more like forcing them. Aaaa they are cooking on the three stone fire, so you drop from the bicycle and stop them. (CoLT member Village 1)

The “surveillance” role of fieldworkers and CoLT members and the oversight of household activities through CAPS suggests that MLW can be viewed as being at the head of a pyramidal research environment where power is produced through a ‘network of relations’ including those between fieldworkers, CoLTs and study participants [49].

### Power and resistance expressed through rumours of blood-taking

Foucault argued that ‘where there is power, there is resistance’ [50]. This theme will explore indications within this study that rumours of blood-taking were used as ‘petty acts of insubordination’ [49], as a challenge to the unequal power of MLW. As concerns about blood taking in clinical trials is common in Malawi as in other sub-Saharan countries [21] a statement was added to the information and consent sheet of the main CAPS trial to state clearly that no blood samples would be collected.

However, an early observation session in Village Five, revealed that such rumours persisted. A passing neighbour began a conversation with the CAPS householder in which she explained how her daughter had participated in the previous ACTia trial despite others warning that researchers would take her child’s blood as they were Satanists. She also said that she had seen JA in the hospital carrying a baby; a sum of MK5000 was mentioned although it was not clear whether this was a gift or payment for the baby. The passer-by had participated in an ancillary CAPS study (the Adult Lung Health Study) but had not been able to enrol in CAPS as she did not have a child under five. Through this exchange, the neighbour introduced the idea of outsiders with nefarious intent, expressed her own ambiguity about research participation and appeared to be communicating that participation in CAPS may be risky. In line with White’s suggestion that vampiric rumours are significant and can provide a way to explore power dynamics [51], further exploration of rumours within CAPS was therefore planned. This episode also illustrates how the “outsider” researcher was enmeshed within the network of rumour and power relations that comprised CAPS.

In the fieldworker FGDs, participants described how concerns and rumours about blood-taking were ongoing within CAPS, despite no obvious mechanism for blood collection. Although some CAPS participants ‘were excited to use the cookstoves’ in contrast to participation in previous trials where blood was taken, fieldworkers shared ongoing concerns about researchers who ‘suck blood’ leading to issues with enrolment (Female fieldworker FGD). The male fieldworkers described how “sensitisation” meetings were organised to try to allay such fears but had limited success with rumours persisting (Male fieldworker FGD).

The female fieldworkers explained that these rumours endured because of the link with ACTia in which there was a straightforward narrative, ‘they take blood from our children and sell it’, (Female fieldworker FGD). They suggested that this resulted in some people proposing that through CAPS a more insidious way of taking blood had been determined, as described in the below discussion:

Facilitator: ‘So when it came to the cookstoves it was built on the problem before do you think?’

Female fieldworker 1: ‘Yes because we are the same people.’

Female fieldworker 2: ‘Yes people said “they have brought another instrument of taking blood”. [They said] they don’t want us to see them directly taking blood but the cookstoves will still be taking blood from us. At the end we will get them, get their blood and use for something else.’

When CAPS participants were asked about rumours within the trial, their responses indicated that risks and benefits of participation were carefully considered and that the source of such rumours often came from non-participants. As described by female CAPS participants:

The negative rumours were huge, [people said] if you take part in this study, they will suck your blood; there is no benefit in the study and those people are satanic. Why would they spend all this money and then give us these items for free! In those times we were a bit frightened. But maybe people said this as they were illiterate, so we just ignored them as MLW can’t be stupid, they can’t get bad things and give them to us? (Female CAPS participant Village 4)

The novelty and expense of cookstoves in this context appeared to feed into the narrative linking cookstoves and blood-taking, with a CoLT member explaining that recipients associated cookstoves with Satanism, with the logic that such a motive was needed to distribute such ‘expensive things’. (CoLT member Village 1). What appears to be suggested here is that the participants envision research participation as a type of exchange. In this case, participants were clear what they were gaining, that is, cookstoves, but unclear about the price they needed to pay. Uncertainty about transactions in the research relationship can lead to suspicion and misunderstanding [19, 48] but more fundamentally reflect ‘asymmetries of power’ [52]. Geissler (2013) suggests that the lack of openness about disparities between researchers and research participants may result in hidden misgivings and tensions which are articulated through occult rumours [46].

Accepting cookstoves from CAPS entailed participants placing their trust in researchers and in MLW. Suggestions that researchers had more to gain than participants are also reflective of the clear power inequity between both groups, that was contested by residents of CAPS villages and mediated by CoLT members and fieldworkers.

## Discussion

Our findings suggest that in the context of scarcity, the food imperative was a key factor in trial participation. Further, that existing knowledge/power trial inequities are strongly amplified by the food imperative, leading to unpredictable effects on trial implementation and outcomes. This analysis suggests that concerns about food reported through an MLW-initiated research process are part of a wider insecurity and dependence on research activities as a survival strategy in a context of ongoing precarity. Taking part in MLW research activities can then be seen as part of the ‘hunter-gatherer’ role of research participants and CoLT members [41]. This indicates that discursive, including economic power inequities, between trial participants and research institutions should be a central consideration within complex interventions in low-income settings.

Kingori (2013) suggests that their position at the ‘coal face of implementing’ research procedures, presents those such as CAPS fieldworkers and CoLT members with ethical challenges as they seek to reconcile ‘numerous competing interests’ [53]. This is evident from CAPS CoLT members descriptions of their intermediary role with demands from CAPS managers for continued compliance on the one hand, and complaints and requests from their village neighbours on the other. For example, requests for cookstoves from those who are ineligible to participate or for broken cookstoves to be fixed.

Both fieldworkers and CoLT members need to balance the interests of community members with the demands of those above them in a hierarchical network of power. Fieldworkers are usually employed just for the duration of a study so like CoLT members they may seek to present themselves as “good” employees to ensure they can access further job opportunities.

Useful insight into the role of CoLT members is provided by Nyirenda et al. (2018) in their exploration of the recruitment of the community advisory groups (CAGS) that preceded the setting up of CoLTs [30]. (All the CoLT members interviewed as part of this study had initially been recruited as CAG members in 2009 when the programme was initiated.) Whereas the stated intention was for CAG members to put forward community concerns to MLW, the authors found that in rural areas such as Chikwawa their role was more instrumental and largely involved tasks related to the implementation of studies [30]. CAG member interviewees described their role as providing a bridge between the community and researchers, but in reality this was largely seen by them as a one-way bridge. That is, they were accountable to researchers but the opposite did not apply, instead ‘they defined their role as a form of employment or hierarchical duty where the orders came from above’ [30].

The results of this study showed that the potential of the mediation role of CAPS fieldworkers and CoLTs to promote the needs and concerns of CAPS participants was also limited by the hierarchical flow of power in the research context. This is not to say that they did not exercise power in their own ways. There are indications that through continued home visits to participants homes, fieldworkers developed a closer relationship with participants that enhanced their own well-being and had the potential to increase trust in MLW as an institution. However, the narratives of fieldworkers and CoLT members indicated that although they sought to contribute to the building of trust and community harmony, this was balanced against their bureaucratic roles in enacting trial processes and pressure to produce “good quality data” through enforcing consistent use of stoves. CAPS fieldworkers and CoLTs exercised discretion and power, but their own autonomy and power was limited by the wider trial context and challenged by trial participants.

As described, the link between cookstoves and concerns of satanic intent and blood-taking arose because CAPS participants linked the trial with previous research where blood was collected as part of the trial process, and MLW as an overarching body. This indicates two important points. First, that from the point of view of Chikwawa residents, CAPS was a continuation of ACTiA and not a separate entity, despite very different fieldwork protocols. This concurs with the findings of Fairhead, Leach and Small (2006) who carried out research in similar large scale Medical Research Council (MRC) trial in The Gambia and concluded that ‘people living in the operational shadow of a research station’ focus on the institution and not on individual trials [19].

Second that trust in MLW was not automatic but subject to question. CAPS participants needed to be able to rebut rumours and in doing so demonstrate their trust in MLW, but this was not a straightforward process. Such concerns could not just be dismissed and were often worrying, and by taking an approach of “we will see for ourselves” CAPS participants appeared to accept that satanic intent was a possibility. The spreading of rumours of blood-taking by non-participants who were not eligible to enrol in the trial (as they had no children under-5) suggests that these actions may have arisen from envy, but the impact was that fieldworkers and CoLT members needed to engage more regularly to encourage enrolment and continued participation.

Decisions about whether to join and remain in CAPS therefore extended beyond the trial itself and depended on trust in CAPS trial intermediaries and in the ongoing relationship with MLW. As Phiri et al. found, trust is integral to the ‘social relations surrounding participants and delivery agents in community-based interventions’ and can be challenged by the pressures on trial intermediaries to achieve trial outcomes [17].

These results also show that CAPS participants took a transactional approach, examining the pros and cons of taking part in the context of the wider relationship with MLW. However, uncertainty about the specific nature of the research bargain, the transactions on each side can lead to misunderstandings and suspicion of researcher motives [19, 48, 52], as shown by the persistence of rumours of blood-taking within CAPS. Kingori et al. (2010) suggest that potential health research participants are presented with a dilemma, with ‘suspicion and fear’ being balanced against benefits and that disruptive rumours of ill intent are a manifestation of such underlying concerns [54].

Mbembe (2001) highlights the potential destructive nature of rumours by describing them as ‘bombs of the poor’ [55]. These results show that within CAPS rumours of blood-taking were persistent and disruptive, causing concern to participants and additional work for fieldworkers and CoLTs. Scott (1985) argues that rumours and ‘malicious gossip’ are intentional and a form of ‘symbolic resistance’ to the more powerful [49], in this case within the research context of CAPS and MLW. Our findings build on the work of others such as Grietens et al. (2014) who suggest that occult rumours in clinical research are ‘a reflection of social injustice and asymmetric power relations’ [21]. This study is also concurrent with Ashforth’s (2015) conclusion that stories of ‘bloodsuckers’ in Malawian villages should remind us of the insecurity of villagers dependant on the largesse of wealthy outsiders, including Whites from overseas [20].

These results also highlight the often-narrow focus of the community engagement that is seen as integral to trials such as CAPS. As Nyirenda et al. (2018) describe in their MLW-based review of approaches to community engagement, such activities are often seen by researchers in practical, instrumental terms, as opposed to the ideal of participatory decision-making throughout the research cycle [56]. Agenyigi et al. (2014) conclude that successful community engagement needs to go beyond promoting greater understanding of a study, and seek to reduce fears and suspicion through the building of trust [47].

## Conclusion

This qualitative analysis has shown the pervasive impact of power relationships on all participants in a large-scale intervention study in a low- income settings, including fieldworkers, community representatives and research institutions. However as suggested by Foucault, power is not simply repressive [24], but is multidimensional and dynamic and can lead to ‘collaboration and transformation’ [57].

The persistence of rumours about blood taking and cookstoves within CAPS illustrates clearly how such narratives are embedded in the wide set of relationships that are present in the research environment [58, 59]. Although such rumours are often reported they can be seen as something that can be managed [17, 21] although precise strategies for doing so are often lacking. Whereas taking White’s view that these “stories” are pointedly told and can be used to explore power dynamics [51], provides a route to a greater understanding of how power and economic inequity hampers strategies to improve global health, including through clean cooking initiatives such as CAPS.

Although it is difficult to explore “intangible” concepts such as power and satanic rumours using Photovoice [60] the methodology facilitated rich insights into the precarity of everyday life in rural Malawi and suggests that transformative approaches must include the least powerful in the research context. However, as Catalani and Minkler (2010) suggest, the highest level of meaningful participation in Photovoice studies requires an emphasis on long term capacity building and action [61]. The idea that participatory visual research gives ‘voice’ to participants has also been critiqued [62].

The use of participatory methods is not a panacea, care must be taken to avoid tokenistic approaches and the process is shaped and constrained by the priorities and power of the actors involved [63] requiring efforts to improve accountability strategies. The approach to the use of Photovoice methodology in this study was informed by Cornwall’s concept of ‘*optimum* participation’, of striving to move along the participatory continuum whenever possible [63]. It was also underpinned by Ponic and Jategaonkar’s (2012) idea (based on the work of Clark et al. [64]) of ‘situated visual ethics’, defined as context relevant and critical means of ethical decision making that take into account that ‘all decisions and actions are framed by various perspectives and embedded in systems of power’ [65].

Our supposition remains that ethical and effective research into cleaner cooking and similar ‘wicked’ [66] complex issues entails a move away from research agendas and processes shaped by more powerful actors and a shift to an emphasis on the priorities of those exposed to the health threats targeted by the SDGs [67]. As suggested by Fox (2015), ‘voice needs teeth to have bite–but teeth may not bite without voice’ [68]. That is, amplifying the voices of marginalised actors in the research process is a crucial step towards improved health and wellbeing for marginalised people. However, providing them with “teeth that bite” requires serious attention to providing the conditions for empowerment, as a critical step towards the shifting of power within the research context.

This leads to the conclusion that interrogation of the existing mechanisms of power is required within organisations, such as MLW, if meaningful change is to be achieved [12]. Emphasis must also be given to the undoing of ‘unknowing’ about the inequalities, of resources and power, that hamper social and scientific advancement in public health initiatives in Africa and other similar low resource settings [46]. Both research participants and research staff such as the CAPS fieldworkers and CoLTs, deserve ‘respect, justice and beneficence’ and this depends on actively tackling power asymmetries [69]. The findings of this study therefore extend beyond CAPS, since they explore and reflect on some of the ‘fault-lines’ that exist within global health initiatives that demand ‘serious self-reflection, courage and action’ [12] to effect the transformative change that reaching the SDGs by 2030 requires.
